# Increased Production and Molecular Weight of Artificial Polyhydroxyalkanoate Poly(2-hydroxybutyrate) Above the Glass Transition Temperature Threshold

**DOI:** 10.3389/fbioe.2019.00177

**Published:** 2019-07-24

**Authors:** Ken'ichiro Matsumoto, Yuki Kageyama

**Affiliations:** Division of Applied Chemistry, Faculty of Engineering, Hokkaido University, Sapporo, Japan

**Keywords:** biobased plastic, polyester, enzymatic synthesis, lactate-polymerizing enzyme, artificial biopolymer

## Abstract

Poly(2-hydroxybutyrate) [P(2HB)] is an artificial polyhydroxyalkanoate (PHA) synthesized using engineered 2-hydroxyalkanoate-polymerizing PHA synthase. In the present study, the effect of temperature on P(2HB) synthesis was investigated. Recombinant *Escherichia coli* harboring PHA synthetic genes were cultivated with 2HB and 3-hydroxybutyrate (3HB) supplementation at varied temperatures ranging from 24 to 36°C for the synthesis of P(2HB) and natural PHA P(3HB), respectively. P(2HB) production and its molecular weight increased considerably at a threshold temperature of 32–34°C. The trend was not observed during the synthesis of P(3HB). Notably, the threshold temperature was close to the glass transition temperature (*T*_g_) of P(2HB) (30°C), while the *T*_g_ of P(3HB) (4°C) was much lower than the cultivation temperature. The results suggest that thermal motion of the polymer chains influenced the production and molecular weight of the obtained polymer. According to the results, the production and molecular weight of PHA drastically changes at the threshold temperature, which is linked to the *T*_g_ of the polymer. The hypothesis should be applicable to PHAs in general, and potentially explains the inability to biosynthesize high-molecular-weight polylactate homopolymer with a *T*_g_ of 60°C.

## Introduction

Polyhydroxyalkanoates (PHAs) are bacterial storage polyesters that are applied as biobased, biodegradable and biocompatible thermoplastics (Sudesh et al., 2011; Zhang et al., 2018). PHAs exhibit a variety of contrasting physical properties, ranging from brittle to flexible and elastic, similar to petroleum-derived plastics (Muhammadi et al., 2015). The physical properties of polymer materials mainly depend on the structures of their monomeric constituents and their molecular weight. The variety of monomer constituents in a polymer is determined largely by the substrate specificity of a PHA synthase (Zou et al., 2017). In contrast, the factors jointly influence the molecular weight of PHA. For example, the concentrations of alcoholic compounds, which induce the chain-transfer reaction of PHA synthase, are negatively correlated with the molecular weight of a PHA (Tomizawa et al., 2010; Hiroe et al., 2015). Class I PHA synthases synthesize higher molecular weight polymers compared to class II PHA synthases. In addition, a high concentration of active PHA synthase protein in cells could decrease the molecular weight of a PHA (Sim et al., 1997). Despite the relatively extensive empirical knowledge above, the underlying mechanisms that influence the molecular weight of PHAs remain poorly understood.

Naturally occurring PHAs are typically composed of 3-hydroxyalkanoates, and the substrate specificity of the PHA synthases influence their carbon numbers (Zou et al., 2017). The biosynthesis of poly(2-hydroxypropionate) (polylactate or PLA), which is structurally similar to PHAs, has attracted the attention of researchers due to its superior properties. However, attempts to polymerize the corresponding monomer substrate, lactyl-coenzyme A (LA-CoA), using wild-type PHA synthases (Valentin and Steinbüchel, 1994) have been unsuccessful. The first lactate (LA)-polymerizing enzyme, which was an engineered PHA synthase from *Pseudomonas* sp. 61-3 with S325T/Q481K pairwise mutation (PhaC1_Ps_STQK), was identified in 2008 (Taguchi et al., 2008). The artificial PHA poly(LA-*co*-3-hydroxybutyrate) [P(LA-*co*-3HB)] copolymers synthesized using PhaC1_Ps_STQK exhibit semi-transparent and flexible properties (Ishii et al., 2017). A key finding was that PhaC1_Ps_STQK efficiently synthesized P(LA-*co*-3HB) copolymer, but not high-molecular-weight PLA homopolymer. Only a low amount (1 wt%) of PLA homopolymer-like polymer (~99 mol% LA) was obtained using PhaC1_Ps_STQK (Matsumoto and Taguchi, 2013). In addition, the molecular weight of the biosynthesized PLA-like polymer was low (10^3^ g/mol in order of magnitude) compared to the molecular weight of typical PHAs (10^4^-10^5^ g/mol in order of magnitude) (Matsumoto and Taguchi, 2013; Ishii et al., 2017). Such correlation between monomer composition and molecular weight has not been reported in natural PHAs (Murugan et al., 2017). Therefore, the inability to biosynthesize PLA has attracted considerable attention among researchers.

We recently measured the *in vitro* LA-CoA polymerization rate using PhaC1_Ps_STQK, and observed that polymerization halted when the molecular weight of the polymerized product, PLA, reached ~3,000 g/mol (Matsumoto et al., 2018b). Consequently, the inefficient PLA synthesis was attributed to the LA-CoA polymerization by PHA synthase. The halting of LA-CoA polymerization was potentially due to low thermal motion of the PLA chain at the reaction temperature (30°C) since the glass transition temperature (*T*_g_) of PLA is 60°C, which suggested that temperature is a potential additional factor influencing PHA molecular weight.

Investigation of the effect of temperature on PLA biosynthesis, however, is a challenge, since PhaC1_Ps_STQK does not maintain the activity at 60°C. Therefore, we focused on the broad substrate specificity of PhaC1_Ps_STQK toward various 2-hydroxyalkanoates (2HAs), such as 2-hydroxybutyrate (2HB) (Matsumoto et al., 2013), glycolate (Matsumoto and Taguchi, 2013), and 2-hydroxy-4-methylvalerate (Mizuno et al., 2018). P(2HB) was selected as the target polymer because the *T*_g_ of the polymer (30°C) is in the cultivation temperature range of *Escherichia coli*. In addition, P(2HB) is transparent and exhibits stretchy properties that differ from those of natural PHAs (Matsumoto et al., 2013) and form homo- and hetero-stereocomplexes with isotactic 2HA polymers (Tsuji and Hayakawa, 2016). Therefore, the polymer potentially expands the applications of PHAs. The aim of the present study was to investigate the effect of temperature on the production and molecular weight of enzymatically synthesized P(2HB).

## Methods

### Plasmids and Culture Conditions

A plasmid, pBS*phaC1*STQK*pct*, bearing the propionyl-CoA transferase (*pct*) from *Megasphaera elsdenii* and *phaC1*_Ps_STQK genes has been constructed previously (Matsumoto et al., 2018a). *E. coli* JM109 harboring pBS*phaC1*STQK*pct* was cultured in 100 mL LB medium containing 2% glucose, 5 mg/mL sodium (*R,S*)-2HB, and 100 μg/mL ampicillin at 24, 26, 28, 30, 32, 34, and 36°C for 48 h. For P(3HB) production, 5 mg/mL sodium (*R,S*)-3HB was used instead of 2HB. The experiments were performed in biosafety level 1 laboratory and by researchers who had undergone biosafety training.

### Polymer Analysis

The polymer was extracted from the lyophilized cells at 60°C using chloroform for 48 h. Chloroform was evaporated and the residual solid was rinsed with methanol to remove lipids. Polymer production and cellular polymer content were determined based on the weights of purified polymers. Molecular weight was determined using gel permeation chromatography as described previously (Taguchi et al., 2008). The polymer structure was confirmed using Proton Nuclear Magnetic Resonance (^1^H NMR) as described previously (data not shown) (Taguchi et al., 2008).

## Results

**Figure 1:**
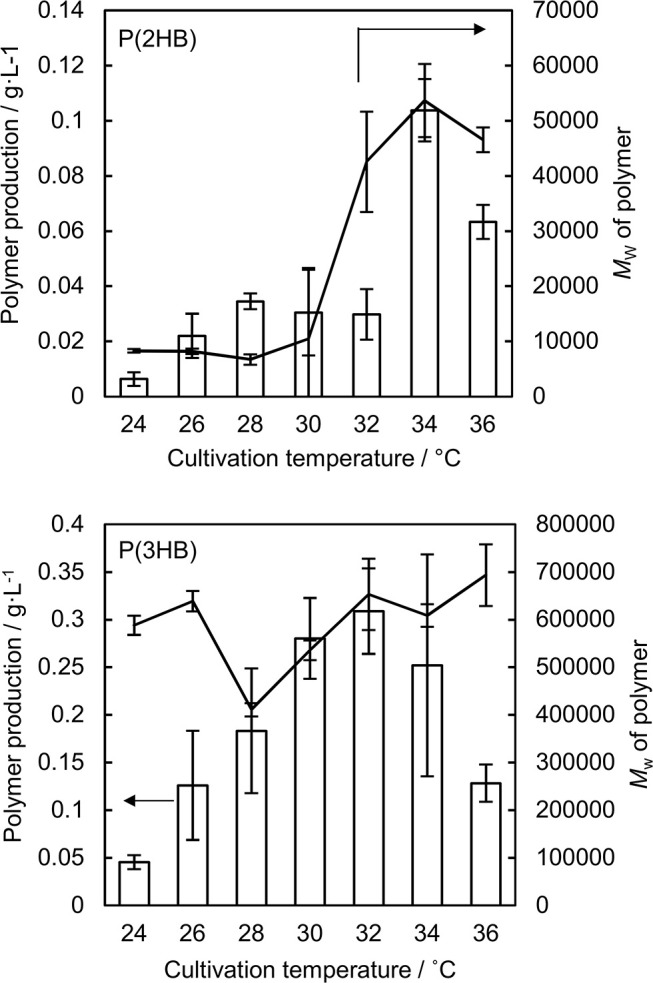
Production and weight-averaged molecular weight of P(2HB) and P(3HB) at a variety of cultivation temperature of engineered *Escherichia coli*. Line graph represents the weight-averaged molecular weight of the polymer. Bar graph represents the polymer production. Data are averages and standard deviations of at least three independent trials.

P(2HB) were synthesized at varied temperatures in the engineered *E. coli* harboring the *pct* and *phaC1*_Ps_STQK. The P(2HB) production increased considerably at 34°C compared to the lower temperatures (Figure 1). In addition, the cellular polymer content also increased markedly from ~1 to 10 wt% at 34°C (Table 1). In contrast, in the 24–32°C range, P(2HB) production and cellular content did not change considerably. The results indicate that high temperature positively influenced P(2HB) production. The weight-averaged molecular weight (*M*_w_) of P(2HB) increased more than 5-fold at 32°C and higher temperatures. The results demonstrate that P(2HB) had a specific threshold temperature, at which the extension of P(2HB) chains was enhanced considerably.

**Table 1:** Cellular polymer content of P(2HB) and P(3HB) in recombinant *Escherichia coli* cultivated under various temperatures.

**Temperature (**°**C)**	**Polymer**			
	**P(2HB)**		**P(3HB)**	
	**Cell dry weight (g/L)**	**Polymer content (wt%)**	**Cell dry weight (g/L)**	**Polymer content (wt%)**
24	3.1 ± 0.1	0.21 ± 0.07	2.4 ± 0.0	1.9 ± 0.3
26	2.5 ± 0.0	0.87 ± 0.32	2.4 ± 0.0	5.3 ± 2.4
28	2.8 ± 0.1	1.2 ± 0.1	3.3 ± 0.4	5.6 ± 1.5
30	2.4 ± 0.1	1.3 ± 0.6	3.5 ± 0.1	9.3 ± 1.9
32	1.8 ± 0.1	1.6 ± 0.5	2.8 ± 0.5	11 ± 1
34	1.0 ± 0.1	10 ± 2	3.1 ± 0.3	8.2 ± 3.4
36	1.1 ± 0.0	5.8 ± 0.5	2.1 ± 0.3	6.0 ± 0.7

The P(2HB) threshold temperature was in the 32–34°C range, which was close to the *T*_g_ of the polymer (30°C). However, reaction rate generally increases based on Arrhenius' equation, and therefore, the enhanced P(2HB) synthesis could have been due to elevated metabolic activity rather than the thermal properties of the polymer chain. Therefore, we tested the effect of temperature on P(3HB) production, which had a *T*_g_ of 4°C.

P(3HB) production and cellular content increased gradually with an increase in temperature up to 32°C, and then decreased at higher temperatures (Figure 1 and Table 1). Notably, P(3HB) molecular weight did not considerably change depending on the temperature (<1.4-fold). Therefore, temperature is a factor influencing PHA synthesis, with greater effects on P(2HB) synthesis compared to P(3HB) synthesis.

## Discussion

The present study demonstrated that the molecular weight and the production of P(2HB) increased markedly at temperatures higher than the threshold temperature. We hypothesized that the phenomenon was due to an increase in the mobility of the polymer chain, which is essential for the progress of polymerization, at the threshold temperature. The threshold temperature is closely related to the *T*_g_ of a polymer. However, *T*_g_ is considered a macroscopic thermal property of the polymer linked to micro-Brownian motion, which is influenced by the intermolecular and intramolecular interactions among high-molecular-weight polymer chains. Conversely, in the initial stage of polymer synthesis by PHA synthase, the polymer chains interact with the enzyme, and potentially, with cytosolic molecules including water. The presence of solvents reportedly influences the *T*_g_ of a polymer (Picker and Hoag, 2002; Kikkawa et al., 2004; Yoshioka and Tashiro, 2004). Therefore, the P(2HB) synthesis threshold temperature was close but not necessarily equal to the *T*_g_ of the polymer.

The hypothesis accounted for the relatively low molecular weight of P(2HB) synthesized at 24–28°C (Figure 1). The *T*_g_ values of a polymer decrease with a decrease in the molecular weight below a certain range (Fox and Flory, 1950). Notably, the *T*_g_ of P(2HB) with a *M*_w_ of 27,000 g/mol was 30°C (Matsumoto et al., 2013), while the *T*_g_ of P(2HB) with a *M*_w_ of 13,000 g/mol was 27°C (Tsuji et al., 2010). P(2HB) with a *M*_w_ of <10^4^ g/mol should exhibit relatively high molecular mobility, and therefore, could be obtained at low temperature conditions.

**Figure 2:**
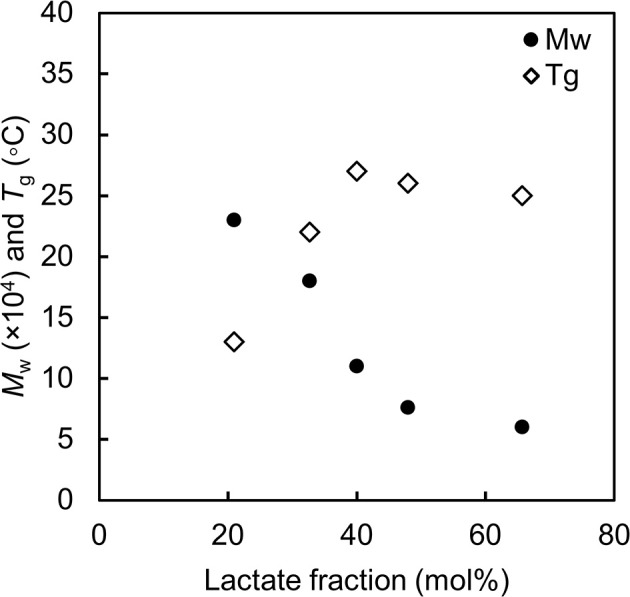
Molecular weight and glass transition temperature of poly(lactate-*co*-3HB) with various lactate fractions. Black circle: weight-averaged molecular weight. White diamond: glass transition temperature of the polymer. The data are from reference by Ishii et al. (2017).

Consistent results have been observed for poly(LA-*co*-3HB) with various monomer fractions (Ishii et al., 2017). Figure 2 summaries the correlation between LA fraction, molecular weight and *T*_g_ of P(LA-*co*-3HB)s synthesized at 30°C. Since P(LA-*co*-3HB) is PLA (*T*_g_ = 60°C) and P(3HB) (*T*_g_ = 4°C) copolymer, the copolymer *T*_g_ should increase with an increase in LA fraction. However, the *T*_g_ values observed for P(LA-*co*-3HB)s reached a plateau below 30°C. The plateaued *T*_g_ were due to a decreasing trend in the polymer molecular weight with an increase in LA fraction. The result indicated that polymer synthesis is limited when the *T*_g_ is higher than the cultivation temperature.

The P(2HB) molecular weight reached a plateau at 32°C and did not increase further with an increase in temperature (Figure 1), which could be because the threshold temperature of P(2HB) represents a type of phase transition similar to glass transition. In general, the phase transition phenomenon refers to a rapid change in the physical properties and structure of molecules under certain conditions. Therefore, based on the hypothesis, polymer molecular weight would not be linearly correlated with temperature. In addition, a reduction in P(2HB) and P(3HB) production at 36°C was potentially due to the thermal instability of PhaC1_Ps_STQK. The enzyme was originally derived from *Pseudomonas* sp. 61-3, which has an optimal growth temperature of 30°C (Abe et al., 1994).

As mentioned above, the lower P(2HB) molecular weight observed at 30°C compared to the molecular weights at higher temperatures were considered due to the limited extension of the polymer chain. However, temperature could also influence the metabolic fluxes involved in monomer supplies. Therefore, the monomer supply rate could influence the polymer molecular weight. According to previously determined kinetic data, PCT exhibits 37.5-fold higher activity in the generation of 2HB-CoA than 3HB-CoA at 30°C (Matsumoto et al., 2018a). In contrast, PhaC1_Ps_STQK activity toward 2HB-CoA was 0.53-fold lower than toward 3HB-CoA (Matsumoto et al., 2018a). Therefore, in comparison with the moderate effect of temperature on P(3HB) synthesis, the 2HB-CoA supply rate was unlikely a P(2HB) synthesis limiting factor at 30°C. Further studies would be required to elucidate the problem.

In conclusion, the results of the present study demonstrated that temperature is a key factor influencing the molecular weight of PHAs. The threshold temperature of the enzymatic polymer synthesis should be close to the *T*_g_ of the polymer. The finding is consistent with our hypothesis that thermal motion of the polymer chain is required for its extension via polymerization. The biosynthesis of some artificial PHAs such as high-molecular-weight PLA, therefore, is limited because the *T*_g_ value of PLA is much higher than the cultivation temperatures. This could be a reason why PLA is not a storage compound in nature, and P(3HB) with a the *T*_g_ much lower than the temperatures appropriate for the growth and development of numerous bacteria is produced by a broad range of bacteria. Other reports have described the biosynthesis of PLA with a molecular weight of 3 × 10^4^ (Jung et al., 2010). The inconsistent results will be discussed elsewhere.

## Data Availability

The raw data supporting the conclusions of this manuscript will be made available by the authors, without undue reservation, to any qualified researcher.

## Author Contributions

KM designed experiments and wrote paper. YK performed experiments.

### Conflict of Interest Statement

The authors declare that the research was conducted in the absence of any commercial or financial relationships that could be construed as a potential conflict of interest.
